# Associations between patient-reported vision impairment in low luminance and vision-related quality of life in intermediate age-related macular degeneration

**DOI:** 10.1038/s41598-025-21210-3

**Published:** 2025-09-26

**Authors:** Jan Henrik Terheyden, Lisa Gittel, Zhichao Wu, Robyn H. Guymer, Robert P. Finger

**Affiliations:** 1 Department of Ophthalmology, University Hospital Bonn, Bonn, Germany; 2 Centre for Eye Research Australia, Royal Victorian Eye and Ear Hospital, East Melbourne, Melbourne, Australia; 3 Ophthalmology, Department of Surgery, The University of Melbourne, Melbourne, Australia; 4 Department of Ophthalmology, Medical Faculty Mannheim, University Hospital Mannheim, University of Heidelberg, Theodor-Kutzer-Ufer 1-3, 68167 Mannheim, Germany

**Keywords:** Vision-related quality of life, Age-related macular degeneration, Patient-reported outcomes, Signs and symptoms, Eye manifestations

## Abstract

Intermediate age-related macular degeneration (iAMD) reduces vision under low-luminance conditions, affecting quality of life (QoL). The Vision Impairment in Low Luminance (VILL) questionnaire captures QoL domains relevant in iAMD but most AMD studies use the generic vision-related QoL measure Visual Function Questionnaire (NEI VFQ). To investigate associations between these generic and specific vision-related QoL instruments, and explore the added value of including the VILL over the NEI VFQ in future iAMD studies, we analysed cross-sectional study data from individuals with iAMD. Associations were investigated using age-adjusted regression models. We included 150 participants (73% women, 74.6 ± 7.8 years). VILL subscale scores were associated with NEI VFQ scores (*p* ≤ 0.014) and explained 62.0% to 67.9% in the variance of the NEI VFQ scores. Ceiling effects were more common in the visual functioning NEI VFQ subscale than in the VILL-Reading subscale (*p* = 0.039), while the emotional well-being scores did not differ signficiantly between the instruments. NEI VFQ scores, VILL-Reading and VILL-Mobility subscale scores were associated with best-corrected visual acuity in either eye. Thus, assessments of vision-related QoL in iAMD using a generic instrument and a low luminance vision-specific instrument yield comparable but not identical results. The VILL has less ceiling effects than the NEI VFQ, suggesting its suitability for iAMD intervention trials and added value over the NEI VFQ in populations with few functional deficits.

**Supplementary Information:**

The online version contains supplementary material available at 10.1038/s41598-025-21210-3.

## Introduction

Age-related macular degeneration (AMD) is a common cause of severe irreversible vision loss across the globe^1^. It leads to a reduction in macular function, first affecting low-luminance and low-contrast vision as well as the ability to adapt to dim lighting conditions, whilst later leading to a reduction in best-corrected vision and a central scotoma^2,3^. This is also reflected in the affected persons’ quality of life (QoL) which continuously decreases from early to intermediate (i) to late AMD^4–6^. Patient-reported outcome measures (PROMs) are systematically developed and validated questionnaire tools that allow reproducible assessment of QoL and are increasingly used in the context of regulatory assessments of pharmaceutical products and medical devices, as quality control measures and in routine care^7^. In ophthalmology and when investigating AMD, the National Eye Institute Visual Function Questionnaire (NEI VFQ) is the most widely used measure to assess vision-related QoL^8^ but its content was not developed to specifically capture the visual difficulties characteristic for early AMD stages, i.e. low-luminance and low-contrast vision as well as difficulties adapting to dark environments^9,10^. The NEI VFQ could thus be prone to ceiling effects when applied in iAMD, as high-luminance visual acuity is often preserved. The Vision Impairment in Low Luminance (VILL) questionnaire has been developed to specifically assess vision-related QoL in the context of domains relevant in iAMD and with requirements for regulatory use of PROMs in mind. It has been shown to be internally consistent, with good test-retest reliability as well as content, construct and predictively valid (e.g., in relation to multiple falls) in the context of AMD^4,11–13^. However, evidence-based information on the relative performance of the VILL when compared to the NEI VFQ and guidance on the selection of targeted PROMs for future iAMD clinical trials is lacking.

In order to investigate the association between the generic vision-related PROM (NEI VFQ-25) and low-luminance-specific PROM (VILL) in iAMD, to establish convergent validity of the VILL, and investigate both questionnaires’ ceiling effects, we have administered the NEI VFQ-25 and the VILL to a cohort of individuals with iAMD. Since the VILL was developed through extensive qualitative research with only AMD patients, we hypothesized that its scores would correlate with general vision-related QoL as assessed by the NEI VFQ-25, while being less susceptible to ceiling effects than the NEI VFQ.

## Methods

### Participants and study procedures

The VILL questionnaire and NEI VFQ-25 were administered cross-sectionally to consecutive individuals with iAMD when they presented to the macular research unit, Centre for Eye Research, Melbourne, Australia. The AMD diagnosis and disease stage were confirmed by grading by two independent graders at each study visits. The study was approved by the Royal Victorian Eye and Ear Hospital Human Research Ethics Committee and adhered to the tenets of the Declaration of Helsinki. Informed consent was obtained from all subjects before the study. The study visits consisted of multi-modal imaging (including optical coherence tomography and colour fundus photography), best-corrected visual acuity (BCVA) testing, and self-administration of PROMs, using paper scores. Inclusion criteria were participants with confirmed iAMD and available data from the NEI VFQ-25 and the VILL questionnaire < 3 months between administrations. Individuals with any eye surgery between study assessments or concurring eye diseases impairing vision were excluded.

### Patient-reported outcome measures

The VILL questionnaire is a 33-item instrument that specifically targets visual deficits under low-luminance and in low-contrast conditions. It has three subscales, i.e. “reading and accessing information” (reading subscale, 17 items), “mobility and safety” (mobility subscale, 12 items) and “emotional well-being” (emotional subscale, 4 items). Its development steps followed regulatory guidelines and included qualitative steps (in–depth interviews, focus group discussions, cognitive debriefings), as well as quantitative steps, where psychometric validity was supported by Rasch models^4,11^. The VILL has been shown to be internally consistent, test-retest reliable, content, construct and prognostically valid in AMD cohorts^4,11^. In addition, the VILL questionnaire has excellent inter-administration mode reliability and prognostic validity related to multiple falls^12,13^.

The NEI VFQ is a common instrument to assess vision-related QoL and was originally developed as a 51-item version, with a subsequent reduction to a 25-item version^9,10^. Its development was originally driven by qualitative work in patient focus groups with age-related cataracts, glaucoma, AMD, diabetic retinopathy, cytomegalovirus retinitis and it has been used in numerous AMD studies since^9,10^. Its traditional sum scoring has been challenged based on psychometric evaluations. Due to this, a Rasch model-scored version, the NEI VFQ-25C, is now used increasingly and has been shown to be reliable and valid^14^. The NEI VFQ-25C has a visual functioning (VF) and a socioemotional (SE) subscale.

### Scoring of patient-reported outcome data

The PROMs were scored following current recommendations for the respective instruments^11,14,15^. The VILL questionnaire was scored based on Rasch models^11^, which belong to the group of latent trait models and assume that all items of a scale represent traits of an underlying measurement construct. The Rasch model fit of the VILL questionnaire was evaluated based on the parameters item fit, person reliability, person separation index and dimensionality^16^. In line with recommendations, items with an infit mean square statistic ≤ 2.0 was considered as non-distortive for the measurement system^17^. A person reliability > 0.8 (0.6) and a person separation index > 2.0 (1.5) were used as indicators of sufficient internal consistency of the scale^11^. Dimensionality was assessed based on the first contrast of a principal component analysis of the residuals, where a contrast < 2.5 eigenvalues was required for unidimensionality^11,18^. For the NEI VFQ, both conventional sum scores and a Rasch model-based scores were used for analysis^10,14^, as the Rasch approach is more state-of-the-art, while conventional sum scoring remains common in many clinical trials. Lastly, the NVQ data were analysed based on Rasch models, as suggested by prior work^15^. Rasch analysis was conducted with Winsteps (version 3.92.1, Chicago, IL).

### Statistical analysis

We investigated associations between NEI VFQ and VILL scores based on Pearson correlation coefficients and multivariable regression analysis. We included NEI VFQ-25 and NEI VFQ-25C scores as dependent variables, and VILL subscale scores, and age as independent variables. Ceiling effects of the PROMs were evaluated and compared between the NEI VFQ-25C and the VILL subscales, using McNemar’s test. Relative ceiling effects were calculated based on the number of data points in the highest 25% of the scale. Furthermore, relative ceiling effects of the PROMs were evaluated based on binary-logistic regression models. In this analysis, we compared neighbouring quartiles of NEI VFQ scores and how these are associated with VILL scores, and vice versa. Lastly, the association between BCVA and QoL outcomes as assessed by the VILL and NEI VFQ-25 was explored based on linear regression models with VILL and NEI VFQ-25C scores as dependent variables. In a backward selection approach, the QoL variables out of the VILL subscale scores and the NEI VFQ-25C global score that were most strongly associated with BCVA were investigated. Statistical analyses were conducted with SPSS for Windows, version 27 (IBM, Armonk, NY). P-values < 0.05 were considered statistically significant.

## Results

We included 150 participants with iAMD, all of whom had completed the VILL and the NEI VFQ-25. The mean age was 75 ± 8 years and the majority of participants were women (Table 1). Most participants identified as Australian (52.7%) or British (18.0%); up to 2% identified as Chinese, Greek, Italian, New Zealander, or Indian. 17% reported another ethnicity, one was unsure, and 5% of ethnicity data were missing. The mean NEI VFQ-25 sum score was 83.7 ± 15.6; the Rasch model-scored vision-related QoL scores are summarized in Table 1. The requirements of the Rasch model were met by the VILL and all items fitted the respective models (Supplementary Table 1) reaching differences between item measures and person measures of 1.43 to 1.82. The internal consistency of the NEI VFQ-25 visual functioning (VF) and socioemotional (SE) subscales was poor (Supplementary Table 1) and the differences between item and person measures of the Rasch-scored NEI VFQ-25 subscales were 1.95 to 2.18. Given these psychometric results, we computed NEI VFQ-25 person measures anchored to a well-established Rasch model derived from a larger population for further analyses.

### Associations between VILL and NEI VFQ scores

**Fig. 1:**
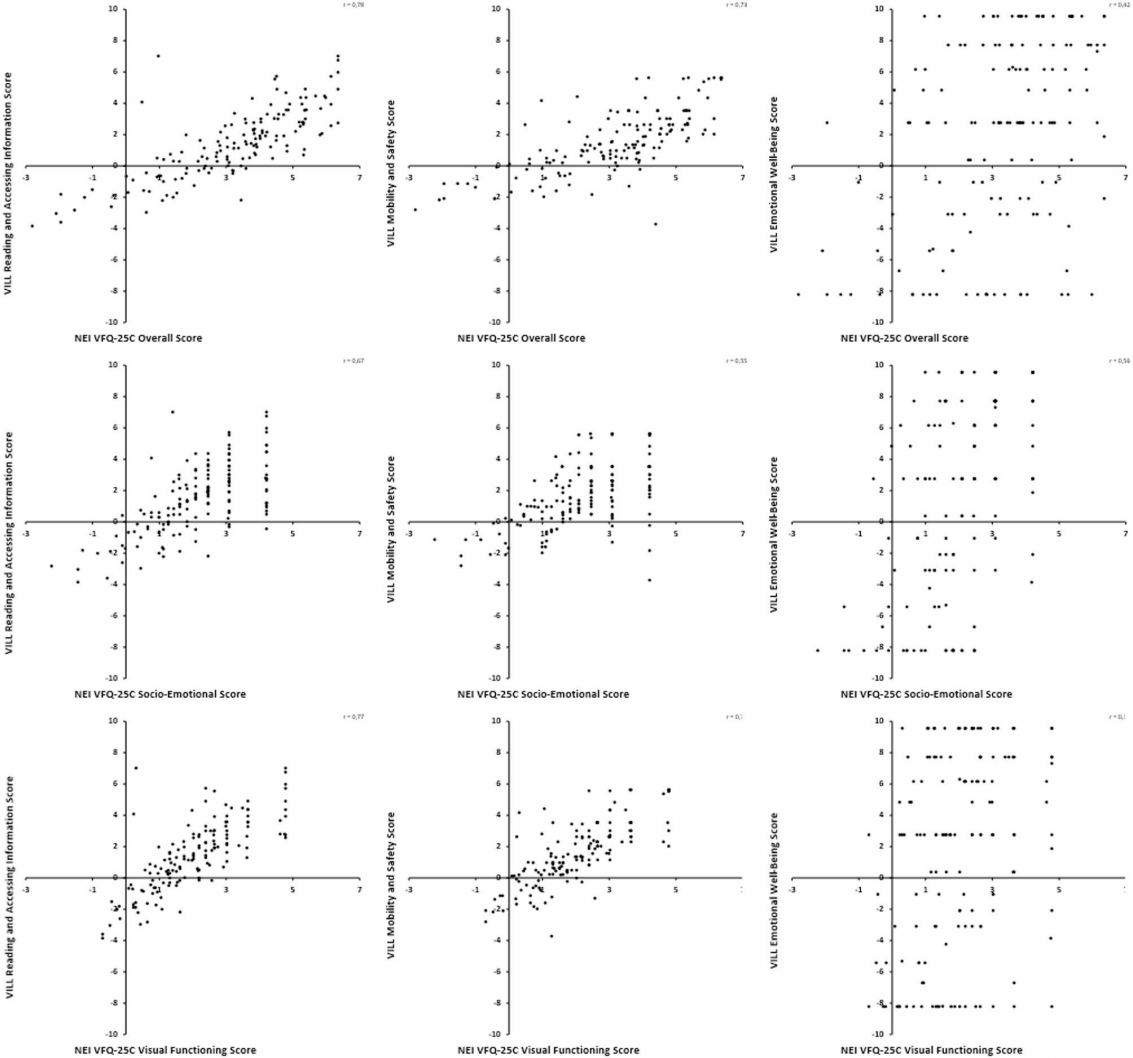
Scatter plot of NEI-VFQ-25C and VILL scores.

Pearson correlation coefficients between NEI VFQ-25C global scores and the VILL-Reading, VILL-Mobility and VILL-Emotional subscale scores were 0.78 [95% confidence interval (CI): 0.71; 0.84], 0.73 [95% CI: 0.64; 0.79] and 0.42 [95% CI: 0.27; 0.54], respectively (Fig. 1). When considering the NEI VFQ-25C subscale scores, the respective correlation coefficients were as follows: 0.77 [95% CI: 0.70; 1.0] and 0.77 [95% CI: 0.71; 1.0] between the NEI VFQ-25C-VF subscale and the VILL-Reading and VILL-Mobility subscales, respectively; and 0.56 [95% CI: 0.46; 1.0] between the NEI VFQ-25C-SE and the VILL-Emotional subscale. Correlation coefficients between the NEI VFQ-25 as per conventional sum scoring and the VILL-Reading, Mobility and Emotional subscales were 0.75 [95% CI: 0.68; 1.0], 0.70 [95% CI: 0.61; 1.0] and 0.37 [95% CI: 0.23; 1.0], respectively.

**Table 1:** Characteristics of the sample of 150 participants with intermediate AMD.

Age [years], mean ± SD	74.6 ± 7.8
Sex, n (%)	
Female	110 (73.3)
Male	40 (26.7)
Best-corrected visual acuity [ETDRS letters], mean ± SD	
Better eye	74.4 ± 16.4
Worse eye	64.7 ± 22.5
VILL-reading	1.4 ± 2.2
VILL-mobility	1.6 ± 2.0
VILL-emotional	1.8 ± 6.2
NEI VFQ-25C-overall	3.2 ± 2.0
NEI VFQ-25C-VF	2.0 ± 1.3
NEI VFQ-25C-SE	2.1 ± 1.4

AMD, age-related macular degeneration; ETDRS, early treatment diabetic retinopathy study chart; SD, standard deviation; SE, socioemotional subscale; VF, visual function subscale; VILL, vision impairment in low luminance questionnaire

When including all VILL subscales into a regression model, the associations with the NEI VFQ-25 and NEI VFQ-25C global scores were statistically significant (Table 2). VILL scores and age explained 62.0% and 67.9% in variance of the scores, respectively; the reverse variance in VILL scores explained by the NEI VFQ and age was in a similar range. When only a subset of NEI VFQ items according to the conventional subscales were included, all VILL subscale scores were significantly predictive of the NEI VFQ-25 subscale general vision (*p* ≤ 0.041). VILL-Reading scores predicted the near activities, distance activities, social functioning, mental health, role difficulties, dependency, peripheral vision NEI VFQ-25 subscale scores (*p* ≤ 0.007). VILL-Mobility scores were predictive of the NEI VFQ subscale distance activities and driving (*p* ≤ 0.002), and VILL-Emotional scores was predictive of the NEI VFQ-25 subscales general health, mental health and driving (*p* ≤ 0.025) when controlling all analyses for age. The NEI VFQ-25 subscales ocular pain and colour vision were not associated with the VILL questionnaire.

**Table 2:** Results of multivariable linear regression analysis including NEI VFQ-25 (conventional scoring) and − 25C (Rasch model-calibrated) scores (dependent variable) and VILL subscale scores (independent variables).

Parameters	NEI VFQ-25		NEI VFQ-25C		NEI VFQ-25C-VF		NEI VFQ-25C-SE	
	β	*p*	β	*p*	β	*p*	β	*p*
VILL-Reading	2.9 [1.6; 4.2]	< 0.001	0.4 [0.3; 0.6]	< 0.001	0.4 [0.3; 0.6]	< 0.001	0.4 [0.3; 0.6]	< 0.001
VILL-Mobility	2.4 [1.0; 3.7]	0.001	0.3 [0.2;0.4]	< 0.001	0.5 [0.3; 0.6]	< 0.001	0.1 [-0.1;0.3]	0.254
VILL-Emotional	0.4 [0.1; 0.7]	0.014	0.1 [< 0.1; 0.1]	< 0.001	< 0.1 [> − 0.1; 0.1]	0.303	0.1 [0.1; 0.1]	< 0.001
Age	− 0.4 [− 0.6; − 0.1]	0.003	>-0.1 [>-0.1; <0.1]	0.316	> − 0.1 [− 0.1; > − 0.1]	0.036	< 0.1 [>-0.01; <0.1]	0.973

NEI VFQ, national eye institute visual function questionnaire; C, rasch model-calibrated score; SE, socioemotional subscale score; VF, visual functioning subscale score; VILL, vision impairment in low luminance questionnaire.

The associations between VILL-Reading and VILL-Mobility subscale scores and the NEI VFQ-25C-VF subscale as well as associations between VILL-Emotional and NEI VFQ-25C-SE were statistically significant (Table 2). In univariable regression, VILL-Reading and VILL-Mobility subscale scores individually explained 59.7% and 57.8% of the variance in NEI VFQ-25C-VF subscale scores. A combination of the two subscale scores in a bivariable model explained 66.7% of the variance in NEI VFQ-25C-VF subscale scores. VILL-Emotional subscale scores explained 25.3% of the variance in NEI VFQ-25C-SE subscale scores.

### Ceiling effects

One percent of participants reached the ceiling of the NEI VFQ and the VILL, respectively, while 2% to 9% of the functional VILL subscale scores (VILL-Reading and VILL-Mobility), 7% of the visual functioning NEI VFQ subscale scores, and 17% of both the VILL-Emotional subcale and the socioemotional NEI VFQ subscale showed ceiling effects. Ceiling effects occurred significantly more frequently in the NEI VFQ-25C-VF subscale compared to the VILL-Reading subscale (*p* = 0.039). No significant differences in the frequency of ceiling effects were observed when comparing the NEI VFQ-25C-VF subscale to the VILL-Mobility subscale or the NEI VFQ-25C-SE subscale to the VILL-Emotional subscale (*p* = 0.508 and 1.0, respectively). Furthermore, based on the distribution of NEI VFQ-25C and VILL subscale data, we hypothesized the presence of relative ceiling effects in the NEI VFQ data, i.e. a clustering of most NEI VFQ scores near the ceiling of the scale. Thirty-five per cent of NEI VFQ-25 responses fell in the highest 25% of the scale (38%, NEI VFQ-25-VF; 71%, NEI VFQ-25-SE), while this applied to 11%, 20% and 37% of VILL-Reading, Mobility and Emotional subscale scores. To further investigate this observation, we analysed the quartiles of the PROM data in our cohort (Supplementary Table 2). VILL-Reading and VILL-Mobility subscale scores were ordered increasingly by NEI VFQ-25C quartiles and yielded significantly higher scores in the highest NEI VFQ-25C quartile over the second highest NEI VFQ-25C quartile (respective odds ratios (OR): 2.049 and 2.276; *p* ≤ 0.001) when controlling for age and BCVA in the better-seeing eye (VILL-Emotional: OR 1.004, *p* = 0.935). However, when the highest VILL subscale quartiles were investigated instead, no significant increase in NEI VFQ-25C scores was found, with an exception in the highest VILL-Emotional quartile and the NEI VFQ-25C-SE subscale scores (Table 3). This supports that the NEI VFQ reached a relative ceiling before the VILL.

**Table 3:** Results of binary-logistic regression analyses investigating The association between VILL subscale quartile (dependent variable) and NEI VFQ-25C scores (independent variables).

VILL subscale quartile membership	2nd over 1st VILL quartile		3rd over 2nd VILL quartile		4th over 3rd VILL quartile	
	OR [95% CI]	*p*	OR [95% CI]	*p*	OR [95% CI]	*p*
VILL-reading quartile						
NEI VFQ-25C	4.0 [2.1; 7.6]	**< 0.001**	2.4 [1.5; 4.1]	**0.001**	1.5 [0.9; 2.4]	0.129
NEI VFQ-25C-VF	4.6 [2.4; 9.0]	**< 0.001**	2.6 [1.6; 4.2]	**< 0.001**	1.2 [0.8; 1.8]	0.382
NEI VFQ-25C-SE	1.8 [1.3; 2.5]	**0.001**	1.9 [1.2; 2.9]	**0.006**	1.9 [0.9; 3.9]	0.084
VILL-mobility quartile						
NEI VFQ-25C	2.5 [1.6; 4.1]	**< 0.001**	3.9 [2.0; 7.6]	**< 0.001**	1.4 [0.9; 2.1]	0.154
NEI VFQ-25C-VF	3.2 [1.9; 5.5]	**< 0.001**	3.0 [1.7; 5.3]	**< 0.001**	1.3 [0.9; 1.9]	0.142
NEI VFQ-25C-SE	1.6 [1.1; 2.1]	**0.007**	2.1 [1.3; 3.5]	**0.003**	1.1 [0.6; 1.9]	0.719
VILL-emotional quartile						
NEI VFQ-25C	1.4 [1.0; 1.8]	**0.025**	1.4 [1.0; 1.9]	**0.033**	1.2 [0.8; 1.7]	0.364
NEI VFQ-25C-VF	1.2 [1.0; 1.5]	0.100	1.3 [1.0; 1.7]	**0.061**	1.0 [0.7; 1.3]	0.788
NEI VFQ-25C-SE	1.5 [1.1; 2.0]	**0.009**	1.4 [1.0; 2.0]	0.068	1.9 [1.0; 3.6]	**0.035**

The quartiles are presented in an increasing order, i.e. The highest VILL quartile includes individuals with The highest vision-related QoL. All models were controlled for age and best-corrected visual acuity of The better-seeing eye.

CI, confidence interval; NEI VFQ, national eye institute visual function questionnaire; SE, socioemotional subscale; VF, visual functioning subscale; OR, odds ratio; QoL, quality of life; VILL, vision impairment in low luminance questionnaire.

*P*-values printed in bold were considered statistically significant.

### Associations between visual acuity and PROMs

Both VILL questionnaire and NEI VFQ-25C scores were significantly associated with BCVA in iAMD (Table 4). In particular, the VILL-Reading and Mobility subscales and the NEI VFQ-25C global scores and VF and SE subscales were associated with high-luminance BCVA, also when controlling the analyses for age. A variable selection approach with backward elimination of variables associated with BCVA in the better eye included all VILL subscale scores and NEI VFQ-25C scores in the final model (R^2^ = 0.319). A similar model targeting predictors of BCVA in the worse eye suggested inclusion of VILL-Reading, VILL-Emotional and NEI VFQ-25C scores in the final model (R^2^ = 0.272).

**Table 4:** Results of univariable linear regression analysis including VILL and NEI VFQ-25C subscale scores (dependent variables) and best-corrected visual acuity (independent variables).

Parameters	VILL-reading		VILL-mobility		VILL-emotional		NEI VFQ-25C		NEI VFQ-25C-VF		NEI VFQ-25C-SE	
	β	*R* ^2^	β	*R* ^2^	β	*R* ^2^	β	*R* ^2^	β	*R* ^2^	β	*R* ^2^
BCVA, better eye	0.066* [0.047; 0.086]	0.240	0.042* [0.023; 0.061]	0.117	0.038 [− 0.023; 0.100]	0.010	0.060* [0.043; 0.077]	0.255	0.071* [0.052; 0.089]	0.272	0.048* [0.031; 0.066]	0.175
BCVA, worse eye	0.046* [0.032; 0.060]	0.220	0.034* [0.020; 0.047]	0.141	0.021 [– 0.024; 0.065]	0.006	0.042* [0.029; 0.054]	0.230	0.050* [0.037; 0.064]	0.263	0.035* [0.023; 0.047]	0.173

BCVA, best-corrected visual acuity; VILL, vision impairment in low luminance questionnaire.

* Statistically significant when controlling for age.

## Discussion

We have demonstrated that vision-related QoL with a focus on low-luminance and low-contrast conditions assessed by the VILL questionnaire is correlated with general vision-related QoL life scores but discriminates between individuals with high self-reported visual functioning slightly better than the NEI VFQ-25. This suggests that both the VILL questionnaire and the NEI VFQ-25 are suitable for assessing patient-reported difficulties in AMD; however, the VILL may be preferable when targeting individuals with higher levels of vision-related QoL due to significantly less ceiling effects. Our study first establishes the convergent validity of the VILL questionnaire based on associations with the NEI VFQ.

Association analyses between VILL and NEI VFQ-25 scores suggest a significant link between “generic” vision-related QoL and low-luminance/low-contrast-“specific” aspects of vision-related QoL. VILL subscale scores similarly relate to visual acuity as the NEI VFQ and explain up to 67% of the variance in NEI VFQ-25C scores, which supports a strong relationship between the metrics. Given the finding that individuals with iAMD and high VILL subscale scores cannot be distinguished from other poorer performers, based on the NEI VFQ-25C, due to a ceiling effect, we suggest including the VILL questionnaire in future studies in iAMD as it is less effected by this ceiling effect. This finding echoes the content domains of the VILL and the NEI VFQ. While the VILL questionnaire was developed for the low-luminance / low-contrast vision domain following health technology assessment guidance for PROM development^19^, the NEI VFQ only includes four night vision items (16% of all items) and was developed on the basis of several conditions, not all of which affect night vision^9^. These general findings are in line with results on the generic vision-related QoL instrument Impact of Vision Impairment (IVI) scale that was not recommended for use in iAMD previously, given its low discriminatory ability when function is only minimally impaired^15^.

The VILL-Emotional subscale provided less granularity compared to the NEI VFQ-25C than the VILL-Reading and VILL-Mobility subscales. In agreement with this, the VILL-Emotional subscale indicated poorer psychometric performance in previous analyses of the MACUSTAR data, which may be related to the lower degree of complexity (only 4 items in VILL-Emotional subscale). Nonetheless, the VILL-Emotional subscale was independently associated with NEI VFQ-25 scores. Future work should also compare the VILL-Emotional subscale with clinical measures of emotional well-being and psychiatric conditions to further establish concurrent validity.

Strengths of our study include the systematic data collection, homogeneity and rigorous phenotyping of the cohort—all with bilateral large drusen (iAMD) and with exclusion of individuals with any other retinal conditions other than AMD–as well as the broad set of PROMs collected from the same cohort. We used the Rasch-scored version of the NEI VFQ (NEI VFQ-25C) rather than the conventionally scored NEI VFQ-25 for the majority of our analyses, which provides an overall score. Given the psychometric weaknesses of the conventional sum scoring of the NEI VFQ, we followed recent recommendations by leading psychometricians in the field^14^. We acknowledge a lack of longitudinal data that will be necessary to better understand the prognostic value of the VILL compared to the NEI VFQ. The ongoing data collection in consecutive patients and in the longitudinal, prospective multi-center study MACUSTAR will further elucidate this^20,21^. An additional limitation of our analysis is the availability of BCVA data as the only functional metric, and the lack of presenting visual acuity data (using existing spectacle / contact lens correction), ideally assessed binocularly, that might have reflected QoL better than monocular BCVA. Since the focus of our study was on the relationship between vision-related QoL PROMs, the association with functional metrics remains to be established further. Lastly, while neither the NEI VFQ nor the VILL measures QoL comprehensively, both are promising PROMs for AMD studies and intervention trials.

Overall, our study supports further use of the VILL questionnaire to assess patient-relevant dimensions in the context of iAMD. VILL questionnaire scores are closely associated with NEI VFQ-25 scores, the most common PROM instrument in the AMD field, but are not as limited by ceiling effects as the NEI VFQ-25, and offers more granularity in individuals with fewer symptoms. Therefore, the VILL questionnaire could help assess the efficacy of treatments that aim to halt or delay disease progression in the future.

## Supplementary Information

Below is the link to the electronic supplementary material.

Supplementary Material 1

## Data Availability

The research data are not publicly available to protect participant privacy but can be requested from the Centre for Eye Research Australia (cera@cera.org.au).
