# Supplementary material for: Associations between patient-reported vision impairment in low luminance and vision-related quality of life in intermediate age-related macular degeneration
Source: Sci Rep. 2025 Sep 26;15:33317. doi: 10.1038/s41598-025-21210-3 (PMC12474979; doi:10.1038/s41598-025-21210-3)
Supplement: Supplementary file 1 — Supplementary Material 1 [file 41598_2025_21210_MOESM1_ESM.docx]

**SUPPLEMENT**

**Vision impairment in low luminance and vision-related quality of life
in intermediate age-related macular degeneration**

**Jan Henrik Terheyden^1^, MD; Lisa Gittel^1^, MSc;
Zhichao Wu^2,3^, BAppSc(Optom), PhD; Robyn H. Guymer^2,3^, MBBS, PhD; Robert P. Finger^1,4^, MD, PhD**

^1^ Department of Ophthalmology, University Hospital Bonn, Bonn, Germany

^2^ Centre for Eye Research Australia, Melbourne, Australia

3 Ophthalmology, Department of Surgery, The University of Melbourne, Melbourne, Australia

^4^ Department of Ophthalmology, University Hospital Mannheim, University of Heidelberg, Mannheim, Germany

**Supplementary Table 1**: Rasch model statistics

|  | VILL-Reading | VILL-Mobility | VILL-Emotional | NEI VFQ-Visual Functioning | NEI VFQ-Socio-emotional | NVQ |
| --- | --- | --- | --- | --- | --- | --- |
| Person Reliability | 0.92 | 0.83 | 0.92 | **0.70** | **0.57** | 0.81 |
| Person Separation Index | 3.50 | 2.19 | 3.39 | **1.53** | **1.15** | 2.10 |
| Misfitting items | 0 | 0 | 0 | 0 | 0 | 0 |
| Residuals PCA, 1^st^ contrast eigenvalue | 2.14 | **2.77** | 1.62 | 2.30 | 1.97 | **3.22** |

NEI VFQ, National Eye Institute Visual Function Questionnaire; NVQ, Night Vision Questionnaire; PCA, principal component analysis; VILL, Vision Impairment in Low Luminance Questionnaire

**Supplementary Table 2**. Summary statistics (mean ± standard deviation) of VILL and NEI VFQ-25C scores by quartiles in the dataset

| **Scale** | **Quartile 1** | **Quartile 2** | **Quartile 3** | **Quartile 4** |
| --- | --- | --- | --- | --- |
| VILL-Reading | -1.39±1.02 | 0.71±0.43 | 2.17±0.43 | 4.33±1.14 |
| VILL-Mobility | -0.90±0.97 | 0.87±0.33 | 2.17±0.42 | 4.23±1.05 |
| VILL-Emotional | -6.45±2.13 | 1.35±1.87 | 6.65±1.11 | 9.56±0.00 |
| NEI VFQ-25C | 0.45±1.26 | 2.88±0.54 | 4.05±0.31 | 5.49±0.53 |

NEI VFQ, National Eye Institute Visual Function Questionnaire; VILL, Vision Impairment in Low Luminance questionnaire
